# Poloxamer 188 Protects Neurons against Ischemia/Reperfusion Injury through Preserving Integrity of Cell Membranes and Blood Brain Barrier

**DOI:** 10.1371/journal.pone.0061641

**Published:** 2013-04-16

**Authors:** Jin-Hua Gu, Jian-Bin Ge, Mei Li, Hai-Dong Xu, Feng Wu, Zheng-Hong Qin

**Affiliations:** 1 Department of Pharmacology and Laboratory of Aging and Nervous Diseases, Soochow University School of Pharmaceutical Science, Suzhou, China; 2 Department of Pharmacology, Nantong University Medical College, Nantong, China; 3 The Second People’s Hospital of Nantong, Nantong, China; New York State Institute for Basic Research, United States of America

## Abstract

Poloxamer 188 (P188), a multiblock copolymer surfactant, has been shown to protect against ischemic tissue injury of cardiac muscle, testes and skeletal muscle, but the mechanisms have not been fully understood. In this study, we explored whether P188 had a protective effect against cerebral ischemia/reperfusion injury and its underlying mechanisms. The *in vivo* results showed that P188 significantly reduced the infarct volume, ameliorated the brain edema and neurological symptoms 24 h after ischemia/reperfusion. In the long-term outcome study, P188 markedly alleviated brain atrophy and motor impairments and increased survival rate in 3 weeks of post stroke period. Additionally, P188 protected cultured hippucampal HT22 cells against oxygen–glucose deprivation and reoxygenation (OGD/R) injury. The ability in membrane sealing was assessed with two fluorescent membrane-impermeant dyes. The results showed that P188 treatment significantly reduced the PI-positive cells following ischemia/reperfusion injury and repaired the HT22 cell membrane rupture induced by Triton X-100. In addition, P188 inhibited ischemia/reperfusion-induced activation of matrix metalloproteinase (MMP)-9 and leakage of Evans blue. Therefore, the present study concludes that P188 can protect against cerebral ischemia/reperfusion injury, and the protection involves multi-mechanisms in addition to the membrane resealing.

## Introduction

Stroke is the second most common cause of death and major cause of disability worldwide [1]. Among various forms of stroke, ischemic stroke is the most common one and occurs when there is an acute interruption of arterial blood flow to the brain. Loss of structural integrity of the cell membranes, due to ischemic damage, is a main cause of necrotic cell death. Most therapeutic strategies have concentrated on inhibiting various steps in signaling process, such as NMDA receptor activation, calcium liberation, and reactive oxygen species production. However, few studies have focused on restoration of the integrity of plasma membranes.

Poloxamer 188 (P188), a biocompatible polymer consisting of two hydrophilic side-chains attached to a hydrophobic center core, has been shown capable of sealing stable defects in cell membranes after various types of injury [2], such as skeletal muscle cell membranes rupture induced by ischemia-reperfusion injury [3], [4], electroporation [5], irradiation [6], and heat damage [7]. In cultured neurons, P188 offered strong protection against excitotoxic injury [8], trauma-induced necrotic and apoptotic cell death [9], [10]. Furthermore, P188 blocked mechanical induced increases in membrane permeability and subsequent cytoskeletal disruption in cultured primary neurons [11], [12]. In vivo delivery of P188 was able to protect neurons from injury induced by spinal cord compression [13], excitotoxicity [14], [15] and acute intracranial hemorrhage [16]. These previous studies have demonstrated the ability of P188 in reducing membrane damage and cell injury in a variety of in vivo and in vitro models.

P188 can come across blood brain barrier [17]. However, when it comes to the effects on the cerebral ischemia, although Colbassani et al had reported that P188 can increase cerebral blood flow in rabbits following ligation of the middle cerebral artery [18], they didn’t explore whether it had a protective effect on ischemic neuronal injury. The loss of integrity of blood brain barrier (BBB) and plasma membrane during ischemic insult is a main pathogenic mechanism leading to brain edema and necrotic death of neurons. Thus maintenance of membrane integrity may attenuate ischemic brain injury. The purpose of this study is to determine if P188 has neuroprotective effects on cerebral ischemia-reperfusion injury and its effects on maintaining the integrity of cell membranes and BBB. The results demonstrated that P188 preserved integrity of plasma membrane and BBB, reduced cerebral ischemic or oxygen-glucose deprivation/reoxygenation-induced damage.

## Materials and Methods

### Mouse Transient Middle Cerebral Artery Occlusion (tMCAO) and P188 Administration

Male ICR mice weighting 25–30 g were purchased from the Center for Experimental Animals of Soochow University (certificate No. 20020008, Grade II). The animal experiments were performed according to the “Principles of Laboratory Animal Care” (NIH Publication 86–23, revised in 1985). The protocol was approved by the Committee on the Ethics of Animal Experiment of Soochow University (Permit Number: 2011-1022-6 ). All surgery was performed under 4% choral hydrate (400 mg/kg) anesthesia, and all efforts were made to minimize suffering. The animals were fasted overnight but were allowed free access to water before surgical procedure. A heating pad and a heating lamp were used to maintain the rectal temperature between 36.5°C and 37.5°C. Through a ventral midline incision, the right common carotid artery, internal carotid artery, and external carotid artery were surgically exposed. The external carotid artery was then isolated and coagulated. A 6-0 nylon suture with silicon coating (Doccol Corporation, Redlands, USA) was inserted into the internal carotid artery through the external carotid artery stump and gently advanced to occlude the middle cerebral artery. Laser-Doppler flowmetry (LDF, ML191 Laser Doppler Blood FlowMeter, Australia) was used to monitor the blockade of cerebral blood flow of middle cerebral artery territory. After 2 h of MCA occlusion (MCAO), the suture was carefully removed to restore blood flow (reperfusion), the neck incision was closed, and the mice were allowed to recover [19]. Those animals of which blood flow recovered up to 80% of pre-ischemia levels were used for further study. The body temperature of mouse was carefully monitored during the post-operation period until the complete recovery from the anesthetic. After the surgery, the animals were housed individually until euthanized. All animals had free access to food and water.

P188 (Sigma-Aldrich) was dissolved in normal saline. In the short-term outcome experiment, mice were administered with P188 (small, medium and large dosages were 0.2, 0.4, 0.8 g/kg body weight, respectively) or vehicle via tail vein 5 min before reperfusion. In the long-term outcome experiment, mice received intravenous injection of P188 (0.4 g/kg) 5 min before reperfusion, and thereafter, animals received daily intraperitoneal (I.P.) injections of either P188 (0.4 g/kg) or vehicle for three weeks post ischemia/reperfusion.

### Evaluation of Infarct Volume, Brain Water Content and Motor Deficits

After 24 hours’ reperfusion, animals were anesthetized with 4% choral hydrate (i.p. injection), the brains were dissected and sliced in a plastic module (Harvard Apparatus, Holliston, USA). Five 1.5 mm thickness sections were obtained and stained with 2% 2,3,5-triphenyltetrazolium chloride (TTC) for 30 min and then fixed with 4% paraformaldehyde. Lesioned areas not stained red with TTC were quantitatively analyzed with Sigma Scan Pro 5. Infarct volume was calculated through slice thickness and the lesioned areas, expressed as a percentage of total hemisphere [19].

Brain water contents were determined 24 h after reperfusion, infarct brain hemispheres were quantified with electronic scale (wet weight) and dried overnight at 105°C in a desiccating oven. The dried brain hemispheres were weighed again (dry weight), and total brain water was calculated according to [(wet weight-dry weight)/wet weight]×100% [20].

The motor deficits of mice subjected to tMCAO were evaluated by an examiner without knowing experimental conditions using the scales as described by Longa et al [21], 0 point, mice behave normally; 1 point, mice cannot fully stretch their left front legs; 2 points, mice turn around into a circle; 3 points, mice fall down to the left side; 4 points, mice cannot move voluntarily, losing consciousness.

### Behavior Test

Wire hanging: Balance and grip strength of mice were assessed with wire hanging test. The wire hanging test apparatus consisted of a steel wire (1 mm) that was stretched between two posts 60 cm above a foam pillow as described [22]. Two days prior to testing, mice were trained to cling to the wire with their forepaws. On the third day, latency to fall from the wire was recorded twice (maximum: 3 min) and results were averaged.

Pole test: The pole test was adapted to Matsuura et al. [23] with minor modifications. A vertical wood pole was covered with tape to create a rough surface. The animal was placed head upward near the top of the pole. The time taken to turn completely downwards (T/turn) and the total time to reach the ﬂoor with all four paws (T/ﬂoor) were recorded. If the animal was unable to turn completely, the time to reach the ﬂoor was also attributed to T/turn. Each animal was tested on 5 trials and the average score was taken as the final pole test score.

### Assessment of Membrane Integrity Using Propidium Iodide Labeling

Propidium iodide (PI) is a 668 Da membrane-impermeable nucleic acid dye that emits bright red fluorescence when it binds to DNA and RNA following cell membrane injury. The protocol of *in vivo* PI staining brain neurons was adapted to a previously published report [24] with minor modifications. PI (Sigma, St Louis, MO, USA) was diluted in normal saline. Briefly, mice were subjected to 2 h MCAO and 23.5 h reperfusion, PI (50 mg/µl, 2 µl) was administered into the ipsilateral cerebral ventricle (coordinates: 0.5 mm posterior to Bregma, 1 mm lateral from midline, and 2.5 mm deep from surface). Mice were deeply anesthetized with choral hydrate and decapitated 30 min after I.C.V. injection of PI. Brains were dissected and stored at −80°C. Brains were cut in the coronal plane on a cryostat. A series of sections (12 µm thick) was collected (150 to 200 µm apart) from anterior to posterior hippocampus from each brain (Bregma −1.90 to −3.00). Cryostat sections were placed on poly-L-lysine-coated glass slides and stored at −80°C. Cryostat sections of the brain were fixed in 100% ethanol for 10 min at room temperature and then allowed to air dry, counterstained with DAPI, and coverslipped with fluoromount (Sigma-Aldrich) for analysis with a fluorescence microscopy (Nikon Eclipse, Tokyo, Japan). Cells labeled with PI or DAPI were counted in the brain regions of interest in all animals from Bregma −1.70 to −3.00 [25]. One field (450×450 µm) per section and 5 to 8 brain sections separated by at least 150 µm in each striatum were chosen for analysis. For hippocampal cell counts, two adjacent dentate gyrus fields were counted from three brain sections separated by at least 150 µm; for CA1, CA2 and CA3 regions, one field in the entire anatomic region of interest was examined in three brain sections. Three animals were analyzed. The effect of P188 on the membrane integrity was calculated as percentage of PI^+^ cells over DAPI^+^ cells.

### Evans Blue Dye Extravasation

The loss of blood-brain barrier (BBB) integrity was assessed by leakage of Evans blue from microvessels after intravenous injection [26]. Evans blue solution (2% in saline, 4 ml/kg body weight) was intravenously administered via the tail vein 22 h after tMCAO. Mice were then transcardially perfused with saline under anesthesia with chloral hydrate 24 h after tMCAO to clear the blood and intravascular Evans blue remaining in the vascular system. After decapitation, forebrains except cerebellum were dissected and divided into ipsilateral and contralateral hemispheres. Each hemisphere was weighed and then homogenized in 50% trichloroacetic acid solution. Following centrifugation at 10000 g for 10 min, supernatants were diluted with ethanol (1∶3). Evans blue was quantified from the absorbance at 620 nm of each supernatant minus the background calculated from the baseline absorbance between 500 and 740 nm and divided by the wet weight of each hemisphere.

### MMP-9 Enzyme Activity and Western Blot Analysis

MMP enzyme activity was assayed by zymogram as described [27]. Briefly, equal amounts (40 µg) of total protein extracts were prepared. After mixing with 2× sample buffer without boiling or denaturing, each sample was separated by Tris–glycine SDS-PAGE with gelatin (1 mg/mL) at 4°C. After electrophoresis was completed, the gel was incubated for 1 h at 25°C in a 2.5% Triton X-100 solution, washed twice with water, for 20 min each, and then incubated overnight at 37°C in a 50 mM Tris–HCl buffer (pH 7.6), containing 0.2 M NaCl, 5 mM CaCl_2_, and 1 µM ZnCl_2_. As a control, duplicate samples were loaded onto another gel that was then incubated in a 50 mM Tris–HCl buffer (pH 7.6), containing 10 mM EDTA to inhibit MMP activity. The gels were fixed with 40% methanol and 7% acetic acid, stained with 0.5% Coomassie blue R-250, and then destained with 10% methanol and 7% acetic acid. Enzyme activity attributed to MMP-9 can be visualized (based on molecular weight) in the gelatin-containing zymograms as clear bands against a blue background.

To determine protein levels of MMP-9 in control and ischemic brains, equal amounts (30 µg) of total protein extracts were prepared. After mixing with 2×sample buffer, each sample was separated by Tris–glycine SDS–PAGE in a reducing condition for MMP-9 and β-actin. After separation, proteins were transferred onto a nitrocellulose membrane. All blots were blocked with 5% nonfat milk in TBS, pH 7.4, containing 0.1% Tween 20 (TBST) for 1 h at room temperature. Then, the membrane was incubated with the primary antibodies against MMP-9 (Santa Cruz) or β-actin (Sigma) diluted in blocking buffer overnight at 4°C. The dilution rates of the primary antibodies were 1∶200 and 1∶5000 for MMP-9 and β-actin, respectively. After washing with TBST, membranes were then incubated with fluorescence secondary antibodies (LI-COR Biosciences), and the signal was read with an Odyssey® Western Blot Analysis system (LI-COR Biosciences, Lincoln, NE, U.S.A.). The signal intensity of primary antibody binding was quantitatively analyzed with Sigma Scan Pro 5 and was normalized to a loading control β-actin.

### Cell Culture and Oxygen–glucose Deprivation and Reoxygenation (OGD/R) Treatment

HT22 murine hippocampal cells were maintained at 37°C in a humidified atmosphere containing 10% CO_2_ in DMEM supplemented with 10% FBS. To mimic ischemic/reperfusion-like conditions in vitro, cell cultures were exposed to glucose deprivation and hypoxia (OGD) for 18 h. Briefly, the cultures were washed three times with Hepes balanced salt solution (HBSS: 140 mM NaCl, 3.5 mM KCl, 12 mM MgSO_4_, 5 mM NaHCO_3_, 1.7 mM CaCl_2_, 0.4 mM KH_2_ PO_4_, 10 mM Hepes), then were placed in an incubator chamber (Billups-Rothenberg, San Diego, CA, USA) aerated with a gas mixture composed of 95% N_2_/5% CO_2_. After the deprivation period, cultures were returned back to the normal culture medium under normoxic conditions for 24 h, corresponding to reoxygenation period (OGD/R). The indicated concentrations of P188 were added to the culture medium 1 h prior to OGD until the end of reoxygenation.

### Cell Viability Assay

HT22 cells were seeded onto 96-well microtiter plates at a density of 1×10^2^ cells/well in 100 µl of DMEM supplemented with 10% FBS. The next day, cells were treated with OGD/R. At the end of reoxygenation period, cell viability was assayed using cell counting kit-8 (CCK8, Dojindo, Tokyo, Japan) according to the manufacturer’s instructions. Briefly, 10 µl of CCK8 solution was added to the culture medium, and incubated for additional 3 h. The absorbance value was determined at 450 nm wavelength with a microplate reader. Alternatively, cell viability was also defined based on the integrity of the cell membrane by measuring the LDH leakage from the cytoplasm into the surrounding culture medium. Briefly, following the OGD/R treatment, cell medium was collected and transferred to a 96-well plate. Then the cells were lysed by freeze-thaw method, and the cell lysate was processed as above. The LDH activities in both the cultures medium and the cell lysate were assayed with cytotoxicity assay Kit (Promega, Madison, USA) according to manufacturer’s protocol. The absorbance value at 490 nm was determined. LDH leakage was calculated as follows: LDH leakage (%) = LDH culture medium/(LDH culture medium+LDH cell lysates)×100%.

### Evaluation of the Effects of p188 on Membrane Integrity of HT-22 Cells

Triton X-100 (Sangon, Shanghai, China), a commonly used detergent in laboratories, can permeabilize unfixed eukaryotic cell membranes. Based on results of the pilot studies, we used 0.03% Triton X-100 to moderately disrupt HT22 cell membranes. After treatment with 0.03% Triton X-100 in DMEM for 5 min, HT22 cells, seeded onto 24-well microtiter plates at a density of 3×10^4^ cells/well, were washed with PBS three times, followed by incubation with PI (15 µM in PBS) for 10 min. after washing steps, P188 (10^−4^ M in PBS) or PBS was added into cell cultures for 10 min. Then cells were incubated in the green fluorescent membrane-impermeant dye SYTOX ® Green (Molecular Probes, 200 nM in PBS) for 10 min. HT22 cells were photographed with a fluorescence microscopy using excitation and emission wave-lengths for PI (535 nm and 617 nm) and SYTOX ® Green (504 nm and 523 nm). Injured cells were defined as PI^+^. Resealed cells were defined as SYTOX ® Green^−/^PI^+^, and cells without resealing as SYTOX ® Green^+^/PI^+^.

### Statistics Analysis

Where appropriate, the data are presented as means ± SD. Statistical analysis was performed with one-way ANOVA followed by Dunnett t-test or performed with log-rank test (survival comparison). GraphPad Prism 5 was used for statistical analysis.

## Results

### P188 Reduces Ischemia/Reperfusion-induced Brain Injury

**Figure 1:**
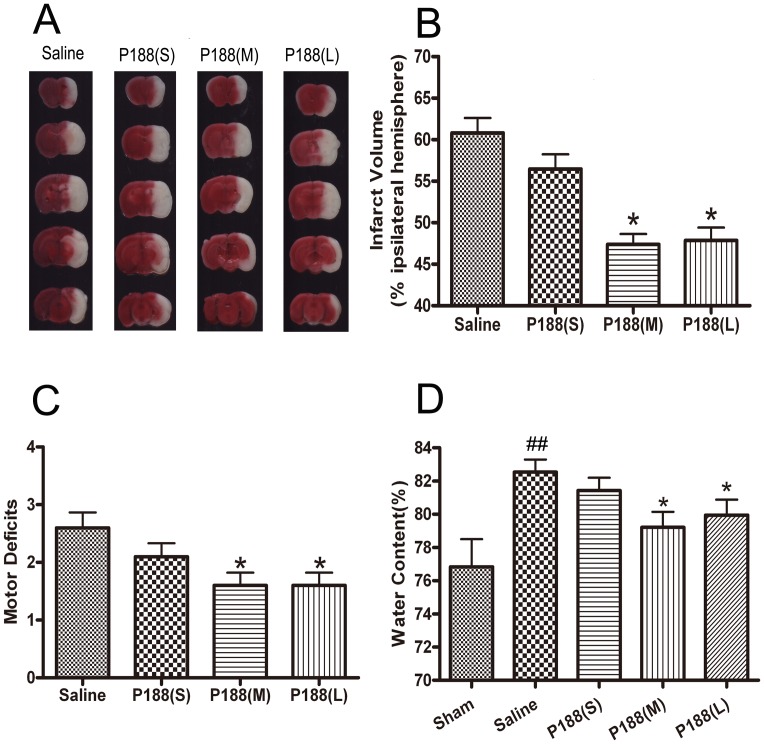
P188 reduces ischemia/reperfusion-induced brain injury. (A) TTC staining of infarct brain regions. (B) Quantitative analysis of brain infarct volume, presented as percentage of the ipsilateral hemisphere. (C) Motor deficits and (D) brain edema. Data are represented as mean ± SD (n = 10 ). *, p<0.05 *vs* the saline-treated group. ^##^, p<0.01 *vs* the sham-operated group. P188 (S) = 0.2 g/kg; P188 (M) = 0.4 g/kg; P188 (L) = 0.8 g/kg.

To determine the effect of P188 on the ischemia/reperfusion injury, P188 (0.2, 0.4, 0.8 g/kg) or vehicle was administered via tail vein 5 min before reperfusion. Lesion size was assessed with TTC staining 24 h after reperfusion. Although a single i.v. injection of a small dose (0.2 g/kg) of P188 failed to reduce the infarct volume, the medium (0.4 g/kg) and large (0.8 g/kg) doses of P188 significantly reduced infarct volume (Fig.1A and B, p<0.05 *vs.* saline-treated group). Remarkable motor behavioral deficits were observed in saline-treated mice. A single i.v. injection of P188 (0.4, 0.8 g/kg) resulted in a significant reduction in motor deficits (Fig.1C, p<0.05 *vs.* saline-treated group). Water content in the mice brains subjected to tMCAO significantly increased compared to sham-operated animals. The medium and large doses of P188 attenuated ischemia-induced increases in brain water content (Fig. 1D; p<0.05 *vs.* saline-treated group). These analyses showed that P188 was able to ameliorate the neurological injury after ischemic attack.

As the large dose could not further enhance the protective effects against brain ischemia, we adopted the medium dosage (0.4 g/kg) in the following long-term outcome experiments.

### P188 Improves Long-term Recovery after Focal Cerebral Ischemia

**Figure 2:**
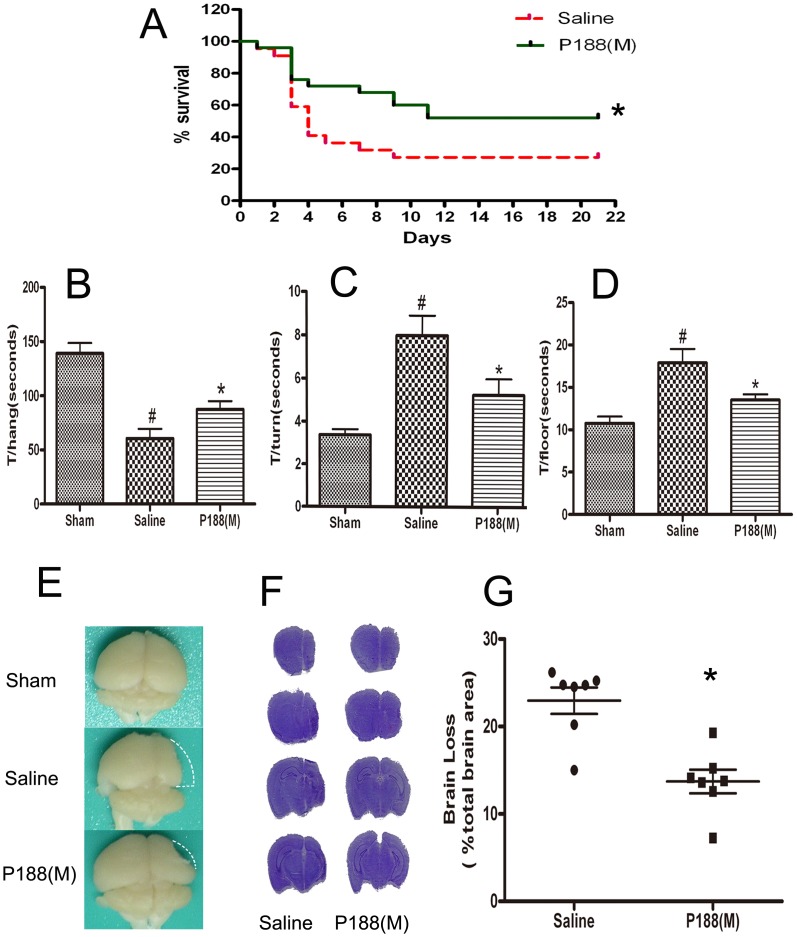
P188 improves long-term functional recovery after ischemia/reperfusion. (A) Post-stroke survival rate three weeks after stroke. *P<0.05, analyzed using log-rank test. (B) Wire hanging test. Time to stay hanging on the wire (T/hang). (C and D) Pole test. Time to turn the head downwards (T/turn). Time to reach the floor (T/floor). ^#^, p<0.05 *vs.* sham-operated group; *, P<0.05 *vs.* saline-treated group. (E-G) Brain atrophy 3 weeks after tMCAO. Brain atrophy was determined using cresyl violet staining. The loss of brain volume was calculated as percentage of brain loss over total brain area of sham-operated mice.

Many neuroprotective strategies have acute effects, but are lack of long-term benefits. Thus, it is of great interest to assess the long-term effects of P188. This study further evaluated the effects of P188 on functional recovery 3 weeks after ischemic brain injury. The findings showed that survival rate was significantly elevated from 27% (n = 51, 14 mice survived) in the saline-treated group to 52% (n = 51, 14 mice survived) in the p188-treated group.(Fig. 2 A, p<0.05).

The wire hanging test revealed a significant difference between P188-treated group and saline-treated group (n = 13 in P188-treated group, n = 14 in saline-treated group, Fig. 2 B, p<0.05). Treatment with P188 for 3 weeks increased the time clung (T/hang) to the wire, compared with saline-treated group. Similarly, the mice from P188 treatment groups took less time to finish a complete turn (T/turn) and to reach the ﬂoor (T/ﬂoor) as compared with mice from saline-treated group (Fig. 2 C and D, p<0.01).

After the behavioral test, 7 mice in each group were randomly chosen to assessed the brain atrophy. The saline-treated mice had apparent brain atrophy in the ipsilateral hemisphere. The mice treated with P188 exhibited less extent of brain atrophy compared to saline-treated mice (Fig. 2 E). The loss of brain volume was analyzed with cresyl violet staining and the results showed 22.96±3.99% brain loss in the saline-treated group, and a 13.71±3.57% brain loss in the P188-treated group (Fig. 2 F and G, p<0.05).

### P188 Preserves the Membrane Integrity after Cerebral Ischemia/Reperfusion Insult

**Figure 3:**
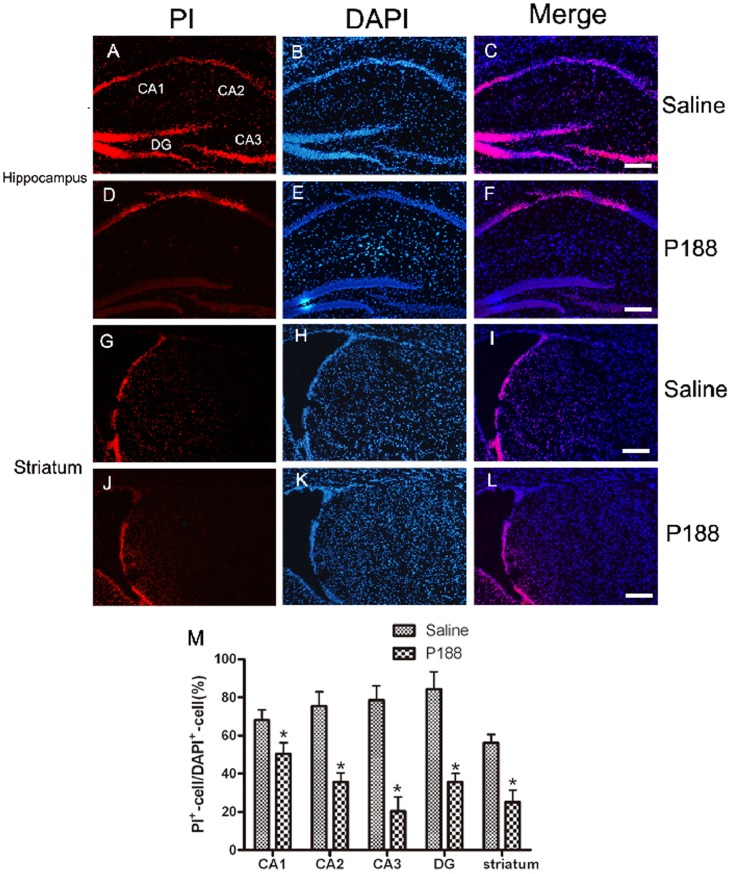
Effects of P188 on the membrane integrity. Cells labeled with PI red fluorescence indicated cells with disrupted membranes (A, D, G and J). Blue fluorescence counterstaining with DAPI indicated total cells (B E H and K). (A) PI-positive cells were abundantly scattered in saline-treated hippocampus, including CA1, CA2, CA3 and DG, while less number of PI-positive cells was found in P188-treated group (D). In the striatal sections, numerous cells were labeled with PI in saline-treated group (G), whereas much less number of PI-positive cells in p188-treated group (J). (M) Quantitative analyses of PI-positive cells in control and P188-treated groups. Data were expressed as percentage of DAPI^+^ cells. *, P<0.05 *vs.* saline-treated group. Bar = 250 µm.

Many studies indicate that PI staining may be a useful tool for assay of membrane integrity in vivo. The present study applied the PI staining method to evaluate the membrane integrity after cerebral ischemia/reperfusion injury. We found that numbers of neurons in hippocampus and striatum labeled by PI significantly increased 23.5 h after reperfusion, indicating disruption of cell membrane (Fig. 3A–C and 3 G–I). P188 treatment 5 min before reperfusion markedly reduced the abundance of PI-positive cells (Fig. 3 D–F and 3 J–L). Quantitative data showed the number of PI-positive cells in injured CA1, CA2, CA3, dentate gyrus and striatum in P188-treated group was significantly less than that in saline-treated group (Fig. 3M). The results indicate that P188 can preserve plasma membrane integrity.

### P188 Reduces Cell Death and Membrane Rupture Induced by OGD/R in HTT-22 Cells

**Figure 4:**
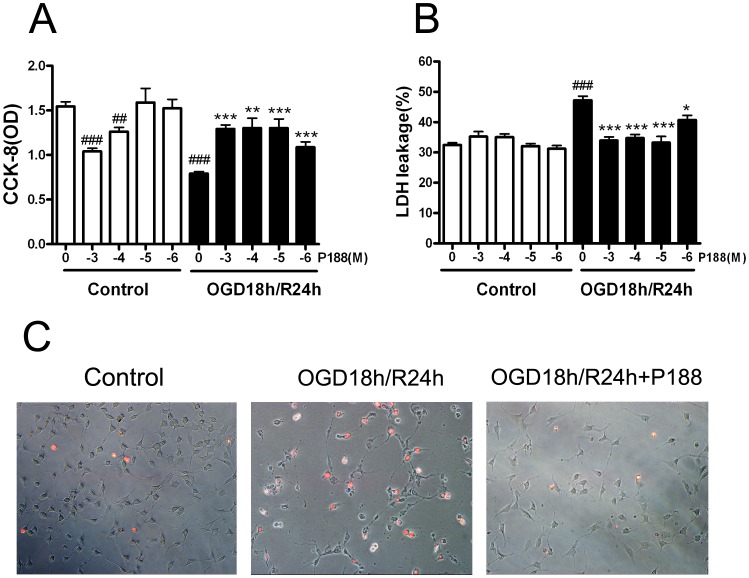
P188 protectes HT22 hippocampal cells against OGD/R-induced cytotoxicity. (A) Cell viability with different concentrations of p188 following OGD 18 h/R 24 h injury or under normal condition (−3, −4, −5 and −6 denoted 10^−3^ M, 10^−4^ M, 10^−5^ M and 10^−6^ M, respectively, ^##^, P<0.01 and ^###^, P<0.001 *vs.* without P188 in control groups, **, P<0.01; ***, P<0.001 *vs.* without P188 in OGD/R condition). (B) LDH leakage induced by OGD18 h/R 24 h injury or in normal condition with different concentrations of p188. ^#^, P<0.05; ^##^, P<0.01 *vs.* without P188 in control groups. *, P<0.05; ***, P<0.001 *vs.* without P188 in OGD/R condition). (C) Representative microphotographs of propidium iodide (PI) fluorescence merged with white bright-light. HT22 cells were under normal condition without P188 or treated with OGD 18 h/R 24 h with or without P188.

After demonstrating the protective effects of P188 on cerebral ischemia/reperfusion injury in mice, we next investigated whether P188 can render protective effects on HT22 hippocampal cells following OGD/R treatment. As shown in Fig. 4 A, P188 could significantly protect against cell injury following OGD/R at all concentrations from 10^−6^ M to 10^−3^ M concentration (p<0.01 *vs.* without P188 in OGD/R condition, n = 6 wells per concentration). It was notable that P188 at 10^−4^ M and 10^−3^ M caused a significant reduction in cell viability in normal condition (P<0.01 at 10^−4^ M and P<0.001 M *vs.* without P188 in control groups, n = 6 wells per concentration). The neuroprotective effects of P188 on cells were further confirmed by LDH leakage assay. Consistent with cell viability, P188 at all concentrations could significantly reduce LDH leakage from HT22 cells induced by OGD/R (Fig. 4 B, p<0.01 *vs.* without P188 in OGD/R condition, n = 6 wells per concentration). However, different from the cell viability, P188 at 10^−4^ M and 10^−3^ M did not increase the LDH leakage of the cell under normal condition. Additionally, we applied PI staining method to assess the membrane integrity following OGD (18 h)/R (24 h) injury. As showed in Fig. 4 C, treatment with OGD (18 h)/R (24 h) greatly increased the proportion of PI-positive cells, while P188 treatment significantly reduced PI-positive cells.

**Figure 5:**
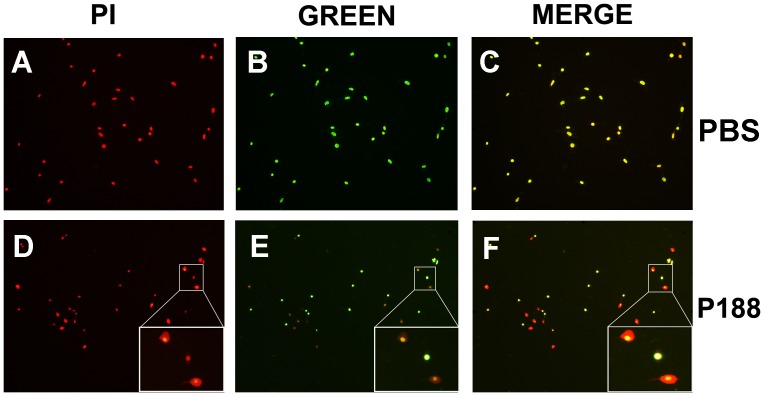
P188 seals the membrane rupture of HT22 cell. Cells stained with PI indicated cells with disrupted membranes (PI^+^, A and D). Cells that remained permeable after P188 or PBS treatment showed green fluorescence (SYTOX® Green^+^, B and E ) or yellow in merged photograph (C and F ). Cell membrane resealed by P188 still showed red fluorescence in merged photograph but no green fluorescence (F).

P188 attenuated the loss of membrane integrity in vivo and in vitro suggest membrane targeting mechanism may be involved in its neuroprotective effects. However, one argue to PI staining results is that membrane damage at 24 h after reperfusion jury in vivo or OGD (18 h)/R (24 h) treatment in vitro is simply indicative of cell necrotic death but not the causative mechanism leading to the observed pathology. Therefore, to explore the membrane-resealing mechanism of P188, we applied Triton X-100, a nonionic detergent, to directly disrupt neuronal membranes. At the same time, two membrane-impermeant fluorescence dyes were sequentially used to observe the changes in membrane integrity. Treatment of cells with 0.03% Triton X-100 robustly disrupted cell membrane (defined as PI^+^, Fig. 5 A and D). Following the treatment with PBS for 10 min, the injury cell membrane remained permeable (defined as SYTOX® Green^+^, Fig 5B or yellow^+^, Fig 5 C). In contrast, P188 treatment prevent the dye SYTOX® Green from penetrating resealed cell membrane (SYTOX® Green^−^, Fig 5E or yellow^−/^PI^+^, Fig 5F), and halation circle was seen around the resealed cells (Fig 5E). Quantity data showed that about 50% of cells were resealed by P188 after Triton X-100 treatment. These results strongly suggest that P188 can seal the membrane rupture induced by Triton X-100.

### Effects of P188 on the Permeability of the BBB and MMP-9 Activity

**Figure 6:**
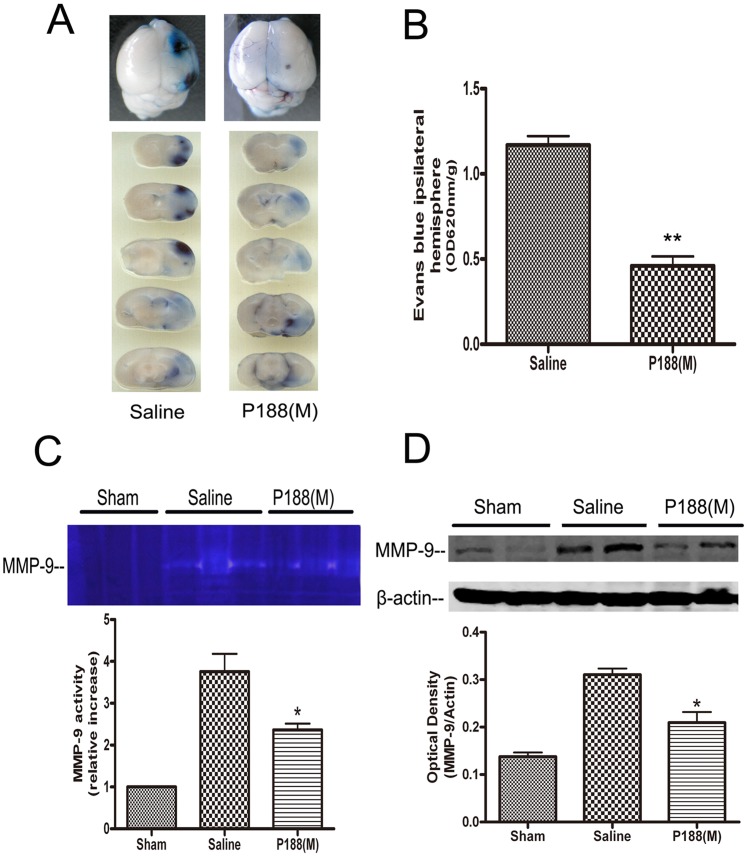
P188 attenuates BBB damage and inhibited protein levels and activity of MMP-9. (A) Evans blue dye extravasation 24 h after tMCAO. The blue staining denoted the areas with increased BBB permeability. (B) Quantification of Evans blue dye extravasation after tMCAO. Bars represent mean ± SE. *, p<0.05 *vs* saline-treated mice (n = 4). (C) MMP-9 activity analyzed by gelatin zymograms. (D) Western blots analysis of MMP-9 protein levels. *, p<0.05 *vs* saline-treated brains (n = 4).

To investigate the effects of P188 on BBB, the mice underwent 2 h MCAO followed by injection with P188, or saline 5 min before reperfusion. Evans blue dye extravasation was analyzed 24 h later. The results showed that administration of P188 (0.4 g/kg) resulted in a significant inhibition of ischemia-induced increase in BBB permeability (Fig 6 A and B, p<0.01). MMP-9 plays an important role in the proteolytic disruption of BBB. To determine whether P188 can regulate MMP-9 expression and activity, mice were administered with P188 or saline 5 min before reperfusion, brain tissue was harvested at 24 h. MMP-9 activity and expression were respectively analyzed by gelatin zymography and immunoblotting. The results showed a less increase in MMP-9 activity in mice treated with P188 than that with saline (Fig. 6C, p<0.05 *vs.* saline-treated group). Similarly, MMP-9 protein levels in P188-treated mice were lower than those in control mice (Fig. 6D, p<0.05 *vs.* saline-treated group).

## Discussion

The potential therapeutic benefits of P188 for treatment of ischemia-reperfusion injury in cardiac muscles [28], testes [29] and skeletal muscles [3], [4] have been previously recognized. Previous studies showed that P188 intravenous injection significantly increased blood flow in severe ischemia areas produced by surgical occlusion of the middle cerebral artery [18]. In this study, we found that P188 (0.4, 0.8 g/kg) significantly reduced infarct volume and water content and ameliorated the neurological symptoms 24 h after reperfusion in the mouse stroke model. Therefore, these studies demonstrated that P188 exerted the protective effects against the brain ischemia-reperfusion induced acute injury.

So far studies about P188 have mainly focused on acute outcome, however, the results about long-term effects on neurological recovery were not consistent. Follis et al showed that intravenous administration of P188 did not prevent paraplegia or improve the long-term neurologic outcome [30]. In contrast, recently daily P188 treatment for 7 days appeared to produce effective long-term neuronal protection in a rat model of intracranial hemorrhage [16]. Therefore, it is of great interest to explore the long-term effects of P188 on cerebral ischemia. Considering the half-life of P188, we administrated P188 once daily for 3 weeks. Here we showed that both histological brain loss and performance on two motor behavioral tasks were significantly improved in P188-treated mice compared to saline group. Furthermore, post-ischemic survival rate of 3 weeks after focal ischemia was also significantly increased. These results demonstrate that daily intraperitoneal injection of P188 can improve the long-term neurologic outcome.

The neuroprotective effects of P188 on ischemia/reperfusion injury were clearly demonstrated in the present study. As expected, the results of *in vitro* experiment also showed that P188 markedly increased the cell viability and reduced the LDH leakage from cytoplasm of HT22 cells following OGD (18 h)/R (24 h) treatment. However, it was notable that 10^−3^ M and 10^−4^ M P188 reduced the cell viability but not increase LDH leakage in normal HT22 cell. We suggest the possibility that high concentration of P188 will markedly change osmotic pressure, therefore inhibit the cell proliferation.

The neuroprotection of P188 has been reported by several other studies, but its underlying mechanisms are not fully understood. Because the chemical structure of P188 is similar to plasmalemma, its insertion into membrane provides an attractive mechanism for restoration of membrane integrity [8]. In this study, we used propidium iodide (PI) to assess membrane integrity *in vivo* [24] and *in vitro*. PI is a membrane-impermeable nucleic acid stain that emits bright red fluorescence when bound to RNA or DNA. When the cell membrane is disrupted, PI leaks into the cells and binds to DNA and RNA. Therefore, only necrotic cells emit red ﬂuorescence [25]. In the present study, mice subjected to transient focal ischemia showed numerous PI-labeled cells in ischemic areas including hippocampus and striatum, but no PI-positive cells were detected in the contralateral hemisphere. P188 treatment significantly reduced the PI-positive cells in hippocampus and striatum area. Similar to *in vivo* results, P188 can also reduce the PI-positive cells following OGD (18 h)/R (24 h) treatment. Because membrane rupture is one of the markers of necrotic cell and there are a great number of necrotic cells following 24 h reperfusion injury or OGD (18 h)/R (24 h) treatment, the PI labeling results may simply indicate P188 can reduce cell necrosis by some undefined mechanisms. Considering the drawback of above results, we used an *in vitro* experimental paradigm that incorporates rapid sequential administration of fluorescent dyes and resealing agents. Just like the membrane damage by electroporation, we applied the Triton X-100, a common detergent in laboratories, to immediately disrupt the cell membrane. The results showed nearly all cell membrane injured by Triton X-100 remained permeable in PBS-treated wells. In contrast, we observed robust membrane resealing with P188 treatment. These results suggest that P188 directly restore membrane integrity following ischemia/reperfusion injury.

In addition to the immediate proposed membrane-resealing mechanism provided by P188, the membrane surfactant may also exert protective effects by other mechanisms. Firstly, The effect of P188 attenuating the brain edema prompted us to explore the integrity of blood brain barrier (BBB) which consists of endothelial cells, the astrocytic foot processes, pericytes, and neurons, and regulates the entry of selected molecules from the blood into the CNS. In pathologic situations such as acute cerebral ischemia, increases in BBB permeability lead to cerebral edema and increased penetration of some molecules usually not permeable to BBB, such as Evans blue. The results showed that P188 could protect BBB against ischemia-induced rupture. Though it is possible that P188 directly repairs the rupture of BBB, we further found that P188 could inhibit MMP-9 protein levels and enzyme activity, which is a key factor involved in BBB disruption. Recent studies reported that Poloxamer regulated the activity of NF-κB. For example, Pluronic P85 (Poloxamer 235) can activate the NF-κB signaling pathway (by inducing phosphorylation of IκB) and upregulate many NF-κB target genes in the muscle cells [31]. Our previous study showed that P188 can inhibit NF-κB signal pathway by reducing the degradation of IκB in the excitotoxicity rat model induced by intrastriatal injection of quinolinic acid [32]. Therefore, we speculate that NF-κB signal pathway may be also involved in the inhibitory effect of P188 on the MMP-9 expression, which is the target gene of NF-κB [33], [34].

### Conclusions

This study demonstrates that P188 provides the long-term protective effects on cerebral ischemia-reperfusion injury *in vivo* and *in vitro.* The neuroprotection offered by P188 involves several mechanisms including preserving BBB impermeability and inhibiting MMP-9 in addition to the membrane resealing.
